# Transcriptomic landscape of airway epithelial repair: Contrasting acute and chronic injury in mustard lung and COPD

**DOI:** 10.1016/j.jgeb.2026.100756

**Published:** 2026-06-24

**Authors:** Masoud Arabfard, Zahra Salehi, Mostafa Ghanei, Sadegh Azimzadeh Jamalkandi

**Affiliations:** a Artificial Intelligence in Health Research Center, Biomedicine Technologies Institute, Baqiyatallah University of Medical Sciences, Tehran, Iran; b Hematology, Oncology and Stem Cell Transplantation Research Center, Research Institute for Oncology, Hematology and Cell Therapy, Tehran University of Medical Sciences, Tehran, Iran; c Chemical Injuries Research Center, Systems Biology and Poisonings Institute, Baqiyatallah University of Medical Sciences, Tehran, Iran

**Keywords:** Airway epithelium, Epithelial repair, Acute and chronic lung diseases, COPD, Mustard lung

## Abstract

**Background:**

Airway epithelial cells play a central role in response to environmental injury and may contribute to progression from acute to chronic respiratory disease. This study investigates transcriptomic changes in airway epithelial cells following exposure to cigarette smoke and mustard gas, and compares acute injury models with chronic disease states, including COPD and mustard lung.

**Methods:**

Five airway epithelial microarray datasets were analyzed, including GSE5372 (mechanical injury; 22 samples, days 0, 7, 14), GSE20257 (COPD; 135 samples: COPD-smokers *n* = 23, healthy-smokers *n* = 53, healthy non-smokers *n* = 59), GSE77942 (smoke exposure; 36 samples), and two mustard-related datasets obtained from the original authors. Differential expression analysis was performed in R (limma) after normalization and filtering, using |FC| ≥ 2 and raw *p* ≤ 0.05. Dataset similarity was assessed by binary scoring and Pearson clustering with pvclust (100 bootstraps, AU ≥ 0.95). Functional enrichment (GO/KEGG) was performed in FunRich with Bonferroni correction, and interaction networks were analyzed using STRING (score ≥ 0.4) and visualized in Gephi.

**Results:**

Comparative transcriptomic analysis of five airway epithelial datasets revealed no differentially expressed genes shared across all conditions (|FC| ≥ 2, raw *p* ≤ 0.05), indicating substantial molecular divergence between acute injury models, epithelial repair processes, and chronic lung disease states. Representative examples included strong upregulation of CYP1B1 in COPD (logFC = 6.10, *p* = 1.31 × 10^−27^) and LRRC63 in mechanical injury (logFC = 4.65, *p* = 1.28 × 10^−4^), alongside marked downregulation of TIMM17A (logFC = −4.62, *p* = 1.46 × 10^−4^) and AURKB (logFC = −2.62, *p* = 1.83 × 10^−16^). Similarity scoring and Pearson-based clustering segregated datasets into distinct injury-response groups with strong bootstrap support (pvclust AU ≥ 0.95). Functional enrichment and network analyses identified coordinated regulation of pathways related to extracellular matrix organization, inflammatory signaling, cell adhesion, and epithelial plasticity (EMT/MET), involving mediators such as FN1, SERPINE1, CAV1, and SNAI2. Temporal analysis of mechanical injury further suggested stage-specific remodeling programs, with extracellular matrix-associated genes enriched at day 14, while chronic smoke exposure and COPD displayed distinct extracellular matrix remodeling patterns. Together, these analyses highlight context-specific transcriptional programs underlying airway epithelial remodeling across diverse respiratory injury conditions.

**Conclusion:**

Airway epithelial injury does not converge on a single transcriptomic signature but instead follows distinct condition-specific programs. Across datasets, EMT/MET-associated gene expression and extracellular matrix remodeling emerged as central processes linking acute injury, epithelial repair, and chronic airway disease. These findings suggest that dysregulated epithelial repair mechanisms may drive persistent airway remodeling in COPD and mustard lung, highlighting EMT-associated pathways as potential targets for future investigation.

## 1 Introduction

The airway epithelium serves as a protective barrier against environmental insults, such as inhaled cigarette smoke, a significant risk factor for various pulmonary diseases. This barrier is formed by intercellular epithelial junctions in the airways, which restrict the entry of pathogens and environmental stressors. Disruption of this epithelial barrier not only exposes the underlying subepithelial layers to harmful airborne factors but also impairs the normal functioning of epithelial cells, potentially contributing to the development of lung diseases. Hence, epithelial cells play a crucial role in the maintenance of lung homeostasis and dynamically interact with the microbiome, pathogens, toxins, and airborne pollutants.^1^ Recent research highlights the importance of epithelial cells as key regulatory cells in both health and disease, acting as potential drivers in the initiation and progression of respiratory disease.^2^, ^3^, ^4^, ^5^, ^6^ Dysfunctional repair mechanisms, in conjunction with exposure to environmental pollutants, play a significant role in the pathogenesis of various chronic lung disorders.

Chronic obstructive pulmonary disease (COPD) is characterized by chronic respiratory symptoms and is diagnosed by spirometry using a post bronchodilation ratio of forced expiratory volume in 1 s (FEV1) to forced vital capacity (FVC) of less than 0.7. The disease results from irregularities of the airways and/or alveoli that cause persistent and progressive airflow obstruction.^7^ COPD remains one of the leading causes of death worldwide and currently ranks third among all causes of mortality. Based on a recent systematic review and meta-analysis, the global prevalence of COPD in people aged 40 years and older was 12.64% using the fixed ratio (FR) criteria and 7.38% using the lower limit of normal (LLN) criteria, respectively.^8^ Specifically, it is projected that COPD-related deaths will reach 4.4 million worldwide by 2040.^9^ Gene–environment interactions are believed to influence normal lung development and epithelial repair processes, potentially contributing to lung damage. The main environmental factors leading to COPD include smoking and inhalation of toxic particles and gases.^10^ Cigarette smoke has been reported to interfere with epithelial repair processes, particularly in the airways. It disrupts airway epithelial barrier function and cell–cell contacts and may reduce the expression of genes involved in epithelial wound healing processes, which may contribute to chronic conditions such as COPD.^11^ Additionally, sulfur mustard (SM) not only exerts acute toxic effects but may also contribute to the development of COPD-like disease as a delayed pulmonary consequence. It is important to note that the COPD observed in the individuals exposed to sulfur mustard exhibits distinct characteristics and is commonly referred to as Mustard Lung (ML).^12^ Although airway epithelial cells play a key role in host defense, homeostasis, and disease progression, limited research has investigated the patterns of epithelial tissue repair and how these processes may differ between acute injury and chronic lung diseases. This knowledge could help improve COPD management because COPD remains a major public health problem.

**Fig. 1:**
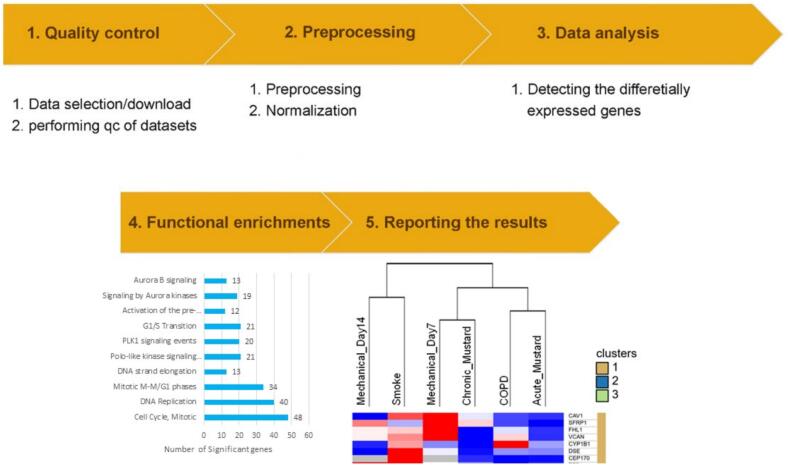
Graphical abstract illustrating the study design and analysis workflow. The flowchart summarizes the sequential analytical pipeline used in this study: **(1)** Dataset selection: transcriptomic datasets of cigarette smoke exposure and COPD were retrieved from the Gene Expression Omnibus (GEO) database, while mustard lung and acute sulfur mustard exposure datasets were obtained directly from the original study authors. **(2)** Data preprocessing and quality assessment: datasets were normalized and evaluated using standard quality control procedures, including distribution inspection and dimensionality reduction plots, to identify potential outliers. **(3)** Differential expression analysis: differentially expressed genes (DEGs) were identified using the *limma* Bioconductor package in R based on fold-change and statistical significance criteria. **(4)** Functional annotation and pathway enrichment: identified DEGs were analyzed to determine associated biological processes and signaling pathways. **(5)** Visualization and interpretation: results were summarized through clustering analyses, heatmaps, and network-based approaches to characterize transcriptional signatures associated with airway epithelial injury and remodeling across cigarette smoke exposure, COPD, and sulfur mustard–related conditions.

This study employs a systems-based approach to characterize transcriptomic patterns in airway epithelial cells under acute and chronic conditions that may reflect alterations in epithelial repair processes. The primary objective of this study is to explore transcriptomic patterns associated with epithelial cell responses to the acute effects of cigarette smoke and sulfur mustard exposure of cigarette smoke and sulfur mustard exposure, while comparing their chronic states observed in COPD and Mustard Lung, respectively. Furthermore, the study aims to examine how transcriptomic profiles in acute and chronic conditions differ from patterns associated with normal epithelial repair. Our findings indicate that acute and chronic conditions are associated with distinct epithelial barrier gene expression patterns and regulatory signatures, highlighting the molecular heterogeneity of lung diseases. Fig. 1 provides a graphical summary of the study design and analysis workflow, outlining the key phases from data source through data processing, analysis, and interpretation to the final output.

## 2 Materials and methods

### 2.1 Data collection

The microarray data were obtained from the Gene Expression Omnibus (GEO) database (ncbi.nlm.nih.gov/gds/). The datasets were selected based on the following inclusion criteria: “Expression profiling by array,” “Epithelial cells,” “COPD,” “Mustard lung injury,” “Cigarette smoke,” and “Human studies.” Profiles that did not focus on either chronic or acute exposure were excluded. Additionally, we excluded all data lacking a time series structure in in vitro studies or staging data in chronic states. Studies involving non-epithelial cells, other treatments, or those with fewer than five samples were also excluded. In total, five series of microarray data were included. Among them, three series GSE5372, GSE20257, and GSE77942 were selected from publicly available sources. Additionally, two data series that were not publicly accessible were identified through literature review and obtained by contacting the respective authors. Detailed information about these datasets is provided in Table 1.

1. The gene expression profile data from GSE5372, referred to as “Mechanical,” consisted of 22 samples of airway epithelium collected from both smokers and non-smokers. This dataset was obtained through bronchoscopy and brushing at three different time points: days 0, 7, and 14 after mechanical injury.^13^ Our study focused on the in vivo repair processes in human airway epithelium, and specifically, we selected day 0 for comparison with day 7, and 14 in our analysis.
2. The dataset GSE20257, referred to as “COPD,” consists of 135 samples obtained from the small airway epithelium of individuals categorized as COPD-smokers (*n* = 23), healthy-smokers (*n* = 53), and healthy non-smokers (*n* = 59). In our analysis, we utilized this dataset to study the chronic state of the epithelium injury.^14^ Specifically, we compared the gene expression profiles across any two states among the three groups (COPD-smokers, healthy-smokers, and healthy non-smokers). To disentangle COPD-specific effects from smoking effects, we performed multiple pairwise comparisons. Specifically, we compared: (1) COPD-smokers vs. healthy-smokers (to identify COPD-associated changes independent of smoking), (2) healthy-smokers vs. healthy non-smokers (to identify smoking-associated changes), and (3) COPD-smokers vs. healthy non-smokers (to assess the combined effect of COPD and smoking).
3. The gene expression profile of GSE77942, named as “Smoke,” comprises 36 samples of human alveolar epithelial cells (A549) that were exposed to cigarette smoke extract at varying concentrations (1%, 5%, and 10%) for different durations (1, 3, and 5 weeks).^15^ According to the original study, CSE media was refreshed every 24 h. The cell cultures were maintained for a week and sub-cultured each time at a density of 6.0 × 10^6 cells per 100 mm dish, until completing 3 and 5 weeks of stimuli, respectively. Human alveolar epithelial cells (A549) at 80% confluence were left in culture medium without serum 24 h before the start of the experiment, followed by incubation with CSE at concentrations of 1%, 5%, and 10% in DMEM plus 1% FBS (fetal bovine serum). In our analysis, we specifically focused on the 10% concentration for a duration of 5 weeks in comparison to the control group. This selection was made to maximize the differences in downstream comparisons, allowing for a clearer identification of the molecular changes and pathways involved in the response to cigarette smoke exposure.
4. The gene expression profiling comprised a total of six samples. This dataset included three patients with Mustard Lung disease in a chronic state who had been exposed to sulfur mustard several years prior to sampling. Additionally, three samples were obtained from unexposed patients.^16^ This dataset was not publicly available and was received directly from the respected investigators. The data from Mustard Lung patients were compared with non-Mustard Lung individuals.
5. The gene expression profiling dataset, known as “Acute_Mustard,” consisted of 8 human primary bronchial epithelial cells (hBEC). These cells were cultured and divided into two groups: four cells were treated with 50 μM sulfur mustard for 18 h, while the other four cells served as the untreated control group. Each group was replicated in quadruplicate. This dataset provided valuable insights into the gene expression response of hBEC to sulfur mustard exposure in an acute setting. This dataset was also not publicly available and was received directly from the respected investigators.^17^

**Table 1:** Summary of the microarray datasets used in the study, including accession number, sample size, condition, and source.

No.	Experimental Variables	GEO accession	Platform	Sample size	References
1	Mechanical injury of airway epithelium	GSE5372	Affymetrix Human Genome U133 Plus 2.0 Array	22	13
2	small airway epithelium of COPD patients	GSE20257	Affymetrix Human Genome U133 Plus 2.0 Array	135	14
3	Human alveolar epithelial cells (A549) exposed to cigarette smoke	GSE77942	Affymetrix Human Gene 1.0 ST Array	36	15
4	Endobronchial biopsy from Mustard Lung patients	Not public	Illumina HT-12 v3 Expression beadchips	6	16
5	Human primary bronchial epithelial cells (hBEC) exposed to sulfur mustard	Not public	Illumina HT-12 v3 Expression beadchips	8	17

Our study faced several limitations that should be acknowledged. First, the number of studies matching the inclusion/exclusion criteria was very limited, which may affect the generalizability of our findings. Additionally, the nature of our research question, encompassing both acute and chronic aspects, posed challenges in obtaining consistent data. The use of different datasets with non-matching time steps or stages further complicated our analysis. Moreover, the lack of access to comprehensive and integrated demographic, clinical, and paraclinical information hindered our ability to fully explore and interpret the data. Nevertheless, we attempted to investigate the trend of changes across different time steps and stages to identify patterns and variations over time, despite the limitations.

### 2.2 | differential expression analysis

The available GEO datasets (GSE5372, GSE20257, and GSE77942) were analyzed using the GEO2R Tool. To ensure data normalization and quality assessment, the built-in functionalities of GEO2R were utilized. For the last two datasets, a preprocessing step was performed, followed by quantitative normalization through quantile-based normalization and logarithmic transformation. Quality control measures, such as histogram plotting and UMAP plots, were used to identify and remove noisy data and outliers; hierarchical clustering in R was also applied. The same preprocessing steps were applied to the non-public datasets, including quantitative normalization through quantile-based normalization and logarithmic transformation. Additionally, histogram plotting and UMAP visualization were implemented to identify and eliminate noisy data and outliers, facilitating effective hierarchical clustering in R.

The process of identifying and removing noisy data and outliers through hierarchical clustering involves several steps. First, the dataset is prepared by scaling and/or normalizing it. Then, hierarchical clustering is applied to group together data points with similar characteristics using distance measures. By analyzing the resulting dendrogram, distinct branches or clusters are identified, and a predetermined dissimilarity or distance threshold is used to detect and eliminate outliers.

Because the included datasets were generated using different microarray platforms, cell types, and experimental designs, they were not merged into a single combined expression matrix. Instead, each dataset was processed and analyzed independently using the limma pipeline with dataset-specific normalization and differential expression analysis. Cross-study integration was subsequently performed at the gene and pathway levels through comparative analysis of differentially expressed genes, clustering patterns, and pathway enrichment results. This strategy avoids direct cross-platform comparisons while enabling identification of shared and condition-specific transcriptomic signatures associated with airway epithelial injury and repair.

Finally, differential expression analysis was performed using the limma Bioconductor package in R. To accommodate differences in sample size, platform, and experimental design across datasets, each dataset was processed and analyzed independently. Linear models were fitted using the lmFit function, followed by empirical Bayes moderation (eBayes) to stabilize variance estimates.

Differentially expressed genes (DEGs) were identified using a combination of statistical and biological criteria. A fold-change threshold of |Fold Change| ≥ 2 was applied to prioritize biologically meaningful expression changes, together with a raw *p*-value ≤0.05. Because several datasets contained relatively small sample sizes, the use of stringent multiple testing corrections substantially reduced statistical power and yielded very few detectable genes. Therefore, raw *p*-values were used at the DEG screening stage to maintain sensitivity, while additional filtering steps were applied to reduce potential noise, including removal of low-expression genes and prioritization of genes consistently meeting the selection criteria across datasets. To avoid cross-platform bias, expression matrices from different studies were not merged. Instead, robust DEGs were identified through intersection of independently generated DEG lists across datasets. This comparative strategy allowed identification of shared transcriptional signals associated with airway epithelial injury and repair while minimizing technical heterogeneity arising from different microarray platforms and sample types.

For genes represented by multiple probes, mean expression values were used to obtain a single gene-level estimate. Given the exploratory nature of the study and the limited sample sizes of several datasets, the results should be interpreted cautiously and considered hypothesis-generating for future experimental validation.

### 2.3 Functional enrichment analysis

Functional enrichment analysis was conducted to explore biological processes using the Gene ontology (GO) database and pathways using in Kyoto Encyclopaedia of Genes and Genomes (KEGG) databases. The analysis was performed using *FunRich* version 3.1.4.^18^ Significant terms and pathways determined by a p-value threshold of ≤0.05 and confirmed by Bonferroni method, were considered for further downstream analysis. This approach allowed for the identification of enriched biological processes and pathways associated with the differentially expressed genes, providing valuable insights into the underlying molecular mechanisms and functional implications of the study.^19^

### 2.4 Data similarity analysis

A comparative analysis was conducted among five datasets, focusing on their identified DEGs and enriched pathways. The similarity among the datasets was estimated using the zero-one scoring method. For the DEG similarity matrix estimation, each gene was assigned a value of one if it was present in the list of DEGs for a particular dataset, and a value of zero if it was not. The same approach was employed to create a pathway similarity matrix.

To visualize the similarities, a correlation-based hierarchical clustering method was implemented using R from the Pearson correlation coefficient with the command *hclust(as.dist(1 – corrx))*, where corrx represents the correlation matrix, and the transformation 1 - corrx is used to convert the correlation values into a distance metric suitable for clustering. This method facilitated the grouping of datasets based on the similarity of their DEGs and enriched pathways. To assess clustering stability, multiscale bootstrap resampling was performed using pvclust (100 iterations, Euclidean distance, Ward.D2). Approximately unbiased (AU) *p*-values (AU ≥ 0.95 indicating robust clusters) were calculated and added to the dendrograms.

### 2.5 Network construction and analysis

The interaction networks pertaining to the epithelial-mesenchymal transition (EMT) and mesenchymal-epithelial transition (MET) associated genes were meticulously constructed utilizing data derived from diverse sources within the STRING database. These sources encompassed text mining, experimental evidence, protein-protein interaction databases, gene fusion data, neighbourhood analyses, and co-occurrence patterns. The establishment of the network involved configuring specific parameters: the network type was designated as a full network, with network edges delineated by confidence measures. A minimum interaction score of 0.4 was deemed necessary for the systematic construction of the network.

Subsequently, the amassed data, networks, and interrelationships between proteins were systematically integrated into Gephi (accessible at https://gephi.org/). This platform facilitated comprehensive visualization and analysis of the intricate network structures. Lastly, the identification of hub genes, pivotal in the network due to their highest degree of connectivity, was accomplished using *network analyzer* packages within the Gephi software.

Network topological properties were calculated using the Network Analyzer tool in Gephi with default parameters. Degree centrality was defined as the number of direct connections (edges) linked to each node, whereas betweenness centrality represented the number of shortest paths passing through a node in the network. Calculations were performed using the default unweighted network settings in Gephi. Unnormalized degree and betweenness centrality values were used to rank nodes in the PPI network, and hub genes were prioritized based on these metrics. The symbols presented in Table 2 indicate the predefined criteria used for hub gene classification.

**Table 2:** Hub genes involved in epithelial-mesenchymal transition (EMT) and mesenchymal-epithelial transition (MET) pathways in acute and chronic conditions.

	Gene symbol	Gene description	Degree⁎	Betweenness Centrality⁎⁎	Mustard gas		Smoke related	
					Acute mustard	Chronic mustard	Smoke	COPD
**EMT**								
1	FN1	Fibronectin 1	36	938.546				
2	CAV1	Caveolin 1	18	359.426	↓	↑		
3	SERPINE1	Serpin family E member 1	14	302.361				
4	CCL2	C-C motif chemokine ligand 2	16	252.435			↓	↑
5	SPARC	Secreted protein acidic and cysteine rich	22	247.403				
6	TAGLN	Transgelin	20	234.995				
7	FBN1	Fibrillin 1	19	177.069	↓	↑	↑	↓
8	VCAN	Versican	12	175.147				
9	WWTR1	WW domain containing transcription regulator 1	4†	165.627				
10	CXCR4	C-X-C motif chemokine receptor 4	13	162.744				
11	GJA1	Gap junction protein alpha 1	13	150.028				
12	SNAI2	Snail family transcriptional repressor 2	15	115.229	↓	↑		
13	FBLN1	Fibulin 1	15	107.331				
14	CD163	CD163 molecule	12	106.589				
15	COL6A2	Collagen type VI alpha 2 chain	16	71.309‡				
16	COL6A1	Collagen type VI alpha 1 chain	16	61.9858‡				
17	CDH2	Cadherin 2	12	30.1926‡				
18	LOXL2	Lysyl oxidase like 2	11	21.9822‡				
**MET**								
1	AGR2	Anterior gradient 2, protein disulphide isomerase family member	16	530.3787	**↓**	↑		
2	ERBB2	Erb-b2 receptor tyrosine kinase 2	14	440.3807			↑	↓
3	FXYD3	FXYD domain containing ion transport regulator 3	5†	374.0393	↑	**↓**		
4	GPX2	Glutathione peroxidase 2	7†	343.7278				
5	PRR15L	Proline rich 15 like	3†	321				
6	CLDN7	Claudin 7	9†	195.0123	↑	**↓**	↑	↓
7	ERBB3	Erb-b2 receptor tyrosine kinase 3	8†	186.0278			↑	↓
8	CBLC	Cbl proto-oncogene C(CBLC)	4†	162.127	**↓**	↑	↑	↓
9	EPCAM	Epithelial cell adhesion molecule	12	149.6462			↑	↓
10	CEACAM5	CEA cell adhesion molecule 5	10	140.9995	**↓**	↑	↓	↑
11	ESRP1	Epithelial splicing regulatory protein 1	6†	131.3194				
12	IRF6	Interferon regulatory factor 6	4†	121.0833	↑	**↓**	↑	↓
13	PLLP	Plasmolipin	3†	113			↑	↓
14	C1orf116	Chromosome 1 open reading frame 116 (c1orf116)	2†	112			↑	↓

⁎ Nodes with >10 edges were selected as hob genes.

⁎⁎ Nodes with betweenness centrality >100 were selected. Also, data is sorted according to betweenness centrality score.

† Nodes with betweenness centrality >100 but degree of <10 edges.

‡ Nodes with betweenness centrality <100 but degree of >10 edges.

## 3 Results

### 3.1 Identification of differentially expressed genes

**Fig. 2:**
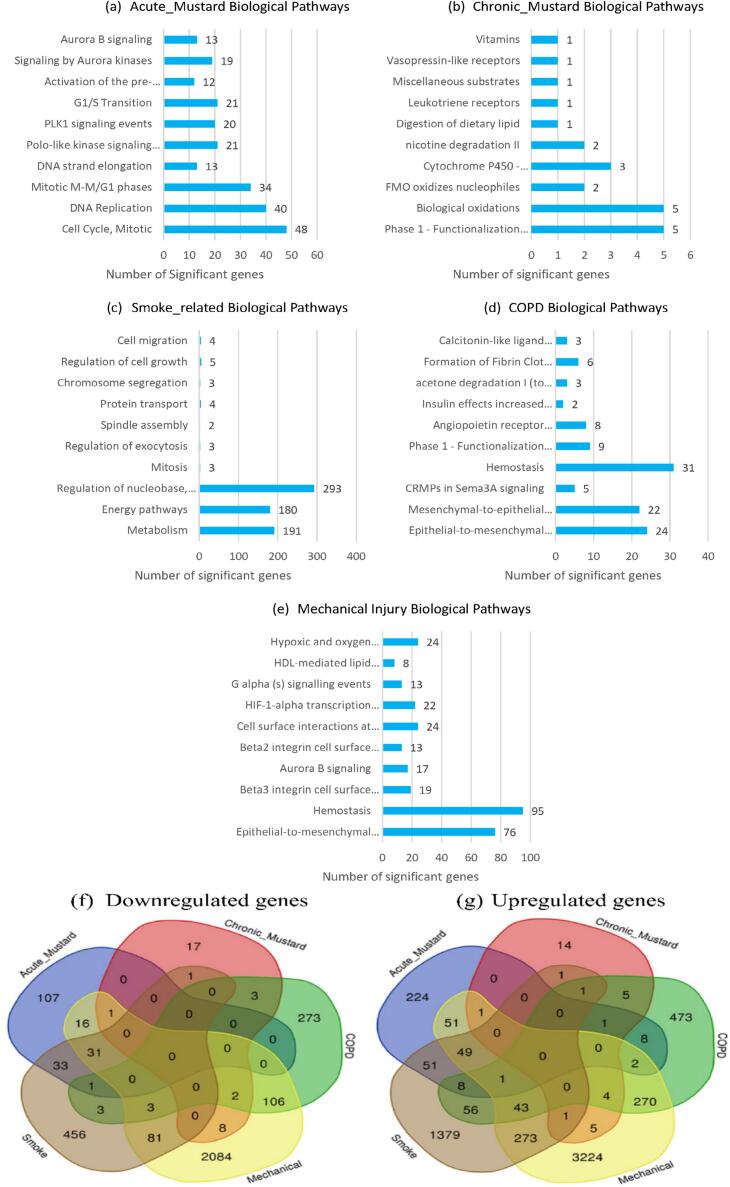
Pathway enrichment and shared differentially expressed genes across airway epithelial injury datasets. Top ten significantly enriched KEGG pathways identified in each dataset: (a) acute sulfur mustard exposure, (b) mustard lung, c) cigarette smoke exposure, (d) COPD, and (e) mechanical injury of airway epithelial cells. Pathways were identified based on enrichment analysis of differentially expressed genes (DEGs) meeting the defined fold-change and statistical significance criteria, (f) Venn diagram illustrating the overlap and disease-specific distribution of significantly downregulated genes across the five conditions, (g) Venn diagram illustrating the overlap and condition-specific distribution of significantly upregulated genes across the five datasets. Each condition is represented by a distinct color, highlighting both shared and unique transcriptional responses among the studied airway epithelial injury models.

To further investigate the specific molecular underpinnings of these conditions, we performed differential gene expression analysis. Detailed analysis parameters, including thresholds for these DEG calculations, are provided in Supplementary File 1. The Venn diagram visually represents the overlap and uniqueness of differentially expressed genes (DEGs) among the disease-related conditions analyzed in the study (Fig. 2: f & g). It shows the number of genes that are commonly and specifically upregulated or downregulated across the different conditions. Additionally, Supplementary File 2 displays the Venn diagram for pairwise comparisons of disease-related conditions, providing a more detailed analysis of the shared and specific DEGs between each pair of conditions.

Among the five datasets analyzed, it was observed that the mechanical injury group had the highest number of dataset-specific downregulated and upregulated genes. Conversely, the chronic mustard group exhibited the fewest specific downregulated and upregulated genes.

Interestingly, no downregulated or upregulated genes were found to be shared among all five conditions, as depicted in Fig. 2 (f and g). This suggests distinct gene expression patterns and regulatory mechanisms associated with each disease condition, reinforcing the heterogeneity of lung-related diseases and their underlying molecular processes.

### 3.2 Identification of significant biological pathways

To identify significant pathways and Gene Ontologies associated with the studied datasets, functional enrichment analyses were conducted. All findings reported in this section are based on adjusted *p*-values. Supplementary File 3 provides comprehensive details of the significant pathways the significant pathways identified for each of the five datasets. In addition, Fig. 2 (a, b, c, d, and e) presents a summary of the results, specifically highlighting the top ten pathways that exhibited significant enrichment across the datasets, allowing for a quick overview of the most prominent biological processes associated with the analyzed conditions.

### 3.3 Estimation of similarities

To elucidate the biological similarities among the five conditions, we performed hierarchical clustering analyses based on both identified biological pathways and DEGs. The statistical robustness of these clustering structures was assessed using multiscale bootstrap resampling (pvclust, 1000 iterations), with all major clusters exhibiting approximately unbiased (AU) *p*-values ≥0.95, confirming strong statistical support.

**Fig. 3:**
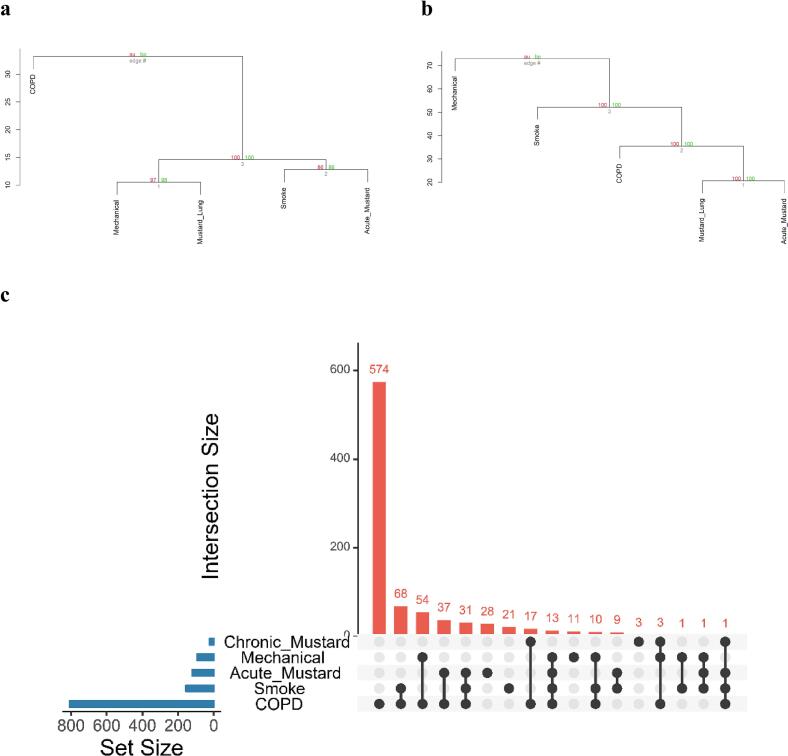
Hierarchical clustering of airway epithelial injury datasets based on pathway and gene expression similarity. (a) Clustering of the five datasets based on enriched biological pathways, illustrating similarity relationships among acute sulfur mustard exposure, mustard lung, cigarette smoke exposure, COPD, and mechanical injury conditions. (b) Clustering based on differentially expressed genes (DEGs), showing an alternative similarity structure among the datasets. In this analysis, the two mustard-related conditions cluster together, followed by COPD and cigarette smoke exposure, while mechanical injury forms a separate branch. c) Bootstrap support values obtained using multiscale resampling (pvclust) are displayed on the dendrogram nodes, where approximately unbiased (AU) values indicate the statistical confidence of each cluster.

Clustering based on biological pathways (Fig. 3a) revealed two main groups: one comprising smoke exposure and acute mustard injury, and another combining chronic mustard injury and mechanical injury. Notably, COPD segregated upstream of these clusters, indicating a distinct biological process profile. In contrast, clustering based on DEGs presented a different pattern (Fig. 3b): the two mustard conditions formed a distinct cluster, followed by a grouping of COPD and smoke exposure. Mechanical injury emerged as an outlier, forming its own cluster. This divergence between pathway- and DEG-based clustering suggests varied molecular underpinnings and highlights mechanical injury as having the least similarity to the other conditions in terms of gene expression (Fig. 3c). These observations underscore the heterogeneity in molecular signatures and biological processes across these disease states, emphasizing the value of multi-faceted analyses for comprehensive disease profiling.

### 3.4 Repair imbalance

**Fig. 4:**
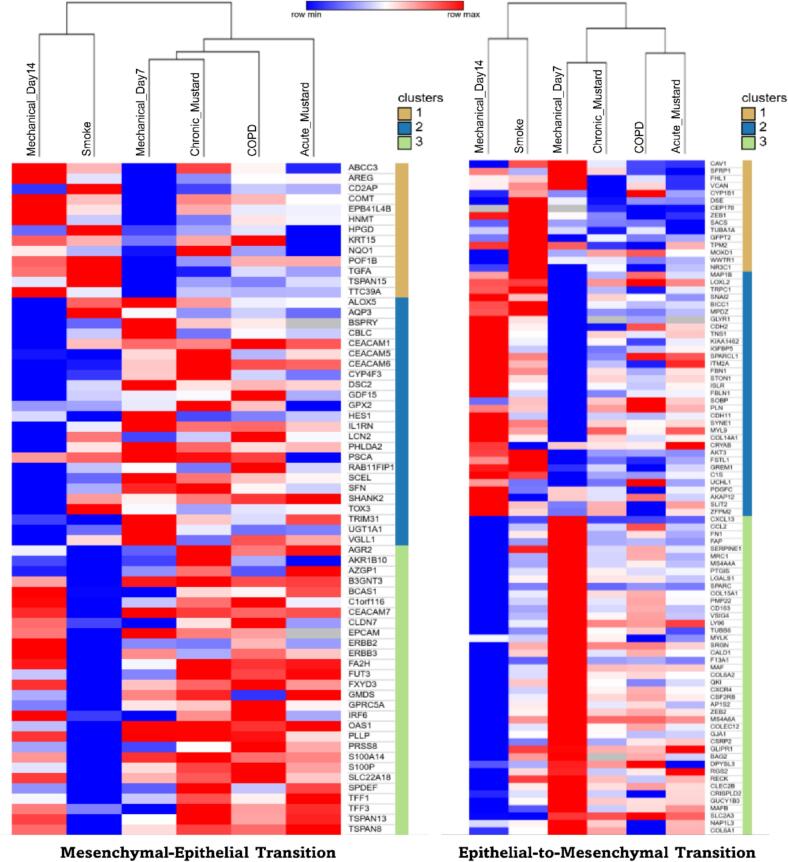
Biclustering heatmap analysis of genes associated with epithelial-to-mesenchymal transition (EMT) and mesenchymal-epithelial transition (MET) pathways. Hierarchical clustering was performed to identify transcriptional modules across the six study conditions (Mechanical_Day7, Mechanical_Day14, Smoke, Chronic_Mustard, COPD, and Acute_Mustard). Genes within the EMT and MET pathways are categorized into three distinct modules (color-coded as indicated in the legend). This visualization highlights the divergent gene expression patterns associated with repair-related processes across different injury models, providing the foundation for the subsequent protein-protein interaction (PPI) network and hub gene analysis.

**Fig. 5:**
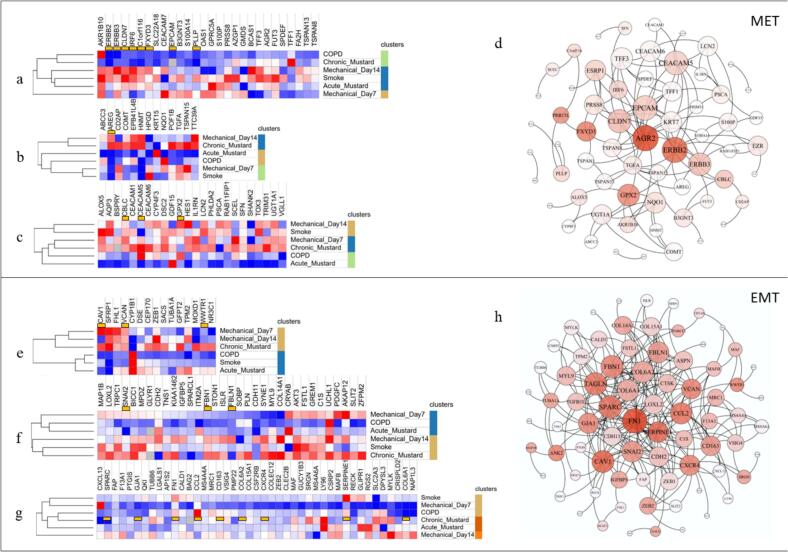
Integrative transcriptional and protein-protein interaction (PPI) network analysis of MET and EMT pathways. (a–c) Hierarchical clustering heatmaps displaying gene expression modules within the Mesenchymal-Epithelial Transition (MET) pathway across the six airway injury conditions. (d) PPI network constructed for MET-associated genes, highlighting key regulatory nodes; node size and connectivity reflect topological importance within the network. (e–g) Hierarchical clustering heatmaps for the Epithelial-Mesenchymal Transition (EMT) pathway, illustrating distinct gene modules across the study groups. (h) PPI network analysis for EMT-associated genes. In both networks (d and h), nodes are color-coded to correspond with the gene modules identified in the respective heatmaps, enabling the identification of central hub genes and their potential role in coordinating repair-associated transcriptional landscapes.

The absence of a consistent differential gene expression signature across all analyzed datasets prompted a transition to a more integrated analytical approach. By performing pathway enrichment analysis and dataset similarity assessments, we identified that responses, while dataset-specific, converged at the pathway level, particularly around processes associated with tissue repair and remodeling. Crucially, gene expression patterns enriched for pathways linked to EMT and MET emerged as a prominent and recurring signal (Supplementary Files 4–7). We therefore utilized EMT/MET-associated transcriptional programs as an interpretative lens to explore the concept of ‘repair imbalance’—defined as dysregulated tissue restoration—and subsequently built protein-protein interaction (PPI) networks focused on these core pathways to uncover potential molecular drivers. These networks, visualized in Fig. 4, Fig. 5, allowed us to cluster genes within each pathway and identify key hub genes and those with the highest betweenness centrality (BC) values, as detailed in Table 2. Supplementary Files 6, 7, and 8 provide the pathway cluster information for EMT and MET, respectively. To further elucidate these complex relationships, a comprehensive gene-pathway-condition network was constructed and illustrated, providing an integrated view of molecular drivers across different conditions (Supplementary Files 9 and 10).

#### 3.4.1 Epithelial-mesenchymal transition

FN1 and SERPINE1 exhibit upregulation in smoke exposure and COPD, but they are decreased in the other groups. Additionally, they are upregulated in smoke exposure and COPD while being downregulated in the remaining groups. VCAN shows downregulation in smoke exposure and COPD compared to the other groups. Interestingly, CCL2 is upregulated in COPD but decreased in the other groups, highlighting a substantial difference between smoke exposure and COPD.

When comparing mechanical injury, we find that GJA1, SPARC, COL6A1, and FBLN1 are upregulated specifically on day 14 of mechanical epithelial injury, whereas they show decreased expression in the other groups. This suggests their potential involvement in the later stages of the healing process following mechanical injury. Furthermore, CD163 is upregulated in both COPD and day 14 of mechanical epithelial injury, while SNAI2 is upregulated exclusively in day 14 of mechanical epithelial injury and chronic mustard exposure. FBN1 is upregulated in smoke exposure, day 14 of mechanical epithelial injury, and chronic mustard exposure. CAV1 is upregulated in day 7 of mechanical epithelial injury and chronic mustard exposure.

COL6A2 and CXCR4 show similar downregulation patterns across all groups, albeit with varying intensities. This indicates a potential role for these genes in the overall response to epithelial injury, regardless of the specific etiology.

**Fig. 6:**
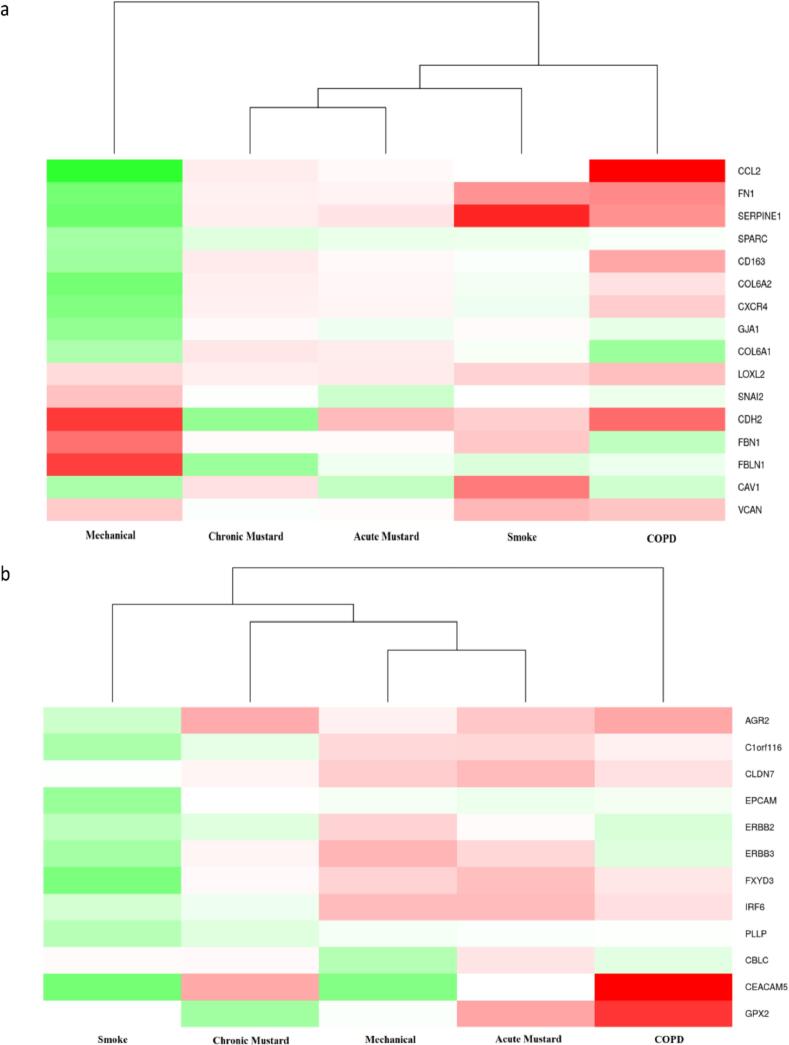
Comparative expression heatmaps of key EMT and MET-associated genes across airway injury models. (a) Differentially expressed genes (DEGs) related to the epithelial-to-mesenchymal transition (EMT) pathway. (b) DEGs associated with the mesenchymal-to-epithelial transition (MET) pathway. The dendrograms illustrate hierarchical clustering of both genes and conditions (Mechanical injury, Chronic Mustard, Acute Mustard, Smoke exposure, and COPD) based on relative expression patterns.

In the investigation of the transition from acute to chronic mustard exposure, WWTR1 is upregulated in both the acute and chronic mustard groups. CAV1 shows different patterns between the two groups, being upregulated in the chronic state and exhibiting a similar pattern in day 7. FBN1 is also increased in the chronic state but not in the acute state, suggesting its involvement in the chronic phase of mustard exposure. Genes SACS, PLN1, and SOBP are specifically upregulated in chronic mustard exposure but not in the other groups, indicating their potential roles in the progression and chronicity of mustard-induced epithelial injury. On the other hand, ITM2A is upregulated only in the acute mustard exposure group, suggesting its specific involvement in the acute response to mustard (Fig. 6a).

#### 3.4.2 Mesenchymal- epithelial transition

In the context of the MET pathway, when comparing the acute and chronic stages of sulfur mustard exposure, we have observed that AGR2, CBLC, and CEACAM5 are decreased, specifically in the acute state. Conversely, FXYD3, CLDN7, and IRF6 are upregulated only in the state of acute.

When comparing the smoke exposure and COPD groups, we have found that ERBB2, CLDN7, ERBB3, CBLC, EPCAM, IRF6, PLLP, and C1orf116 are upregulated after smoke exposure. However, it is worth noting that CEACAM5 is upregulated as well, indicating its potential involvement in both smoke exposure and COPD.

Furthermore, we have observed specific downregulation of AKR1B10 and TFF1, non-hub genes, exclusively in the chronic mustard group. Conversely, SHANK2 is upregulated only in the chronic mustard group. These differential expressions of non-hub genes provide insights into their potential roles in the chronic state of mustard exposure and its associated epithelial injury (Fig. 6 b).

## 4 Discussion

In lung-related diseases such as COPD, smoke exposure, and chronic mustard exposure, the epithelial cells, which play a crucial role in protecting against environmental insults and self-renewal, fail to provide an effective self-repair mechanism. Therefore, it is of great importance to assess the shared and distinct biological pathways and processes between these lung-related diseases and the natural mechanisms of epithelial cell renewal.^5^, ^6^ Despite various studies having been conducted on the biological mechanisms of lung-related diseases, the pathways associated with dysfunctional epithelial repair for such diseases have not been thoroughly investigated and compared.

Unlike previous studies that focused on individual conditions, we analyzed both acute and chronic states of COPD, Mustard Lung, sulfur mustard exposure, and cigarette smoke, revealing distinct and shared pathways. For instance, we identified unique DNA repair pathways activated by sulfur mustard exposure that are not observed in Mustard Lung or mechanical injury. Additionally, our observations regarding differential expression of genes involved in telomere maintenance pathways in smokers, alongside the expression patterns of genes associated with MET and EMT signaling pathways, highlight the complexity of epithelial cell responses.

Our findings demonstrate that smoke exposure and acute sulfur mustard exposure, as triggers of epithelial cell injury, elicit distinct and unique pathways. Specifically, sulfur mustard exposure induces several DNA repair response pathways as observed in Table 3. It appears that sulfur mustard causes significant damage to the genome, resulting in bulky and double-stranded DNA breaks which are aligned with previous reports.^20^ To counteract this, epithelial cells activate various DNA repair signaling pathways to prevent genome instability. These pathways include the Global-genome Nucleotide Excision Repair (NER), and double-strand DNA break repair pathway. Notably, two sensory systems of Global-genome NER, namely GG-NER (independent of transcription) and TC-NER (dependent on transcription by RNA polymerase II complex), are activated in response to sulfur mustard exposure.^21^ Interestingly, the double-stranded DNA repair pathway is also observed in COPD and smokers. However, we did not observe any DNA repair signaling pathway in the Mustard Lung or mechanical injury of epithelial cells. These findings highlight the distinct molecular responses and pathways activated in different lung-related diseases and acute epithelial cell injuries. The identification of specific DNA repair response pathways associated with sulfur mustard exposure provides insights into the mechanisms underlying its genotoxic effects. Moreover, the absence of DNA repair pathways in Mustard Lung and mechanical injury suggests the involvement of different mechanisms in these conditions.

**Table 3:** Summary of condition-specific pathways and their functional classification. Pathways identified in each condition were grouped into broader biological categories based on established functional annotations and evidence from literature. Each pathway was assigned according to its known involvement in specific cellular processes. This classification facilitates interpretation of the biological context and highlights the functional relevance of the pathways identified across the studied conditions.

Classification	Pathways	Smoke	COPD	Mechanical	Mustard Lung	Acute Mustard
Acetylcholine	Muscarinic acetylcholine receptors			**1**		
	Acetylcholine neurotransmitter release cycle			**1**		
Biosynthesis	Asparagine biosynthesis I					**1**
	Zymosterol biosynthesis	**1**				
	Pyrimidine deoxyribonucleotides de novo biosynthesis					**1**
	Superpathway of geranylgeranyl diphosphate biosynthesis I (via mevalonate)	**1**				
	Guanosine nucleotides de novo biosynthesis					**1**
	Derine biosynthesis	**1**				
	Dermatan sulfate biosynthesis (late stages)	**1**				
Cell cycle	Meiotic synapsis	**1**				
	Polo-like kinase mediated events	**1**				
	Mitotic metaphase/anaphase transition					**1**
	DNA replication initiation					**1**
	Polymerase switching					**1**
	Removal of the Flap Intermediate					**1**
	RSK activation	**1**				
EMT and MET	C-src mediated regulation of Cx43 function and closure of gap junctions			**1**		
	p130Cas linkage to MAPK signaling for integrins			**1**		
	Regulation of gap junction activity			**1**		
	GRB2:SOS provides linkage to MAPK signaling for Intergrins			**1**		
	Recycling pathway of L1			**1**		
	NICD traffics to nucleus	**1**				
	Notch-HLH transcription pathway	**1**				
	Notch receptor binds with a ligand				**1**	
	Receptor-ligand binding initiates the second proteolytic cleavage of Notch receptor				**1**	
	Signaling by TGF beta	**1**				
DNA damage response	ATM mediated phosphorylation of repair proteins					**1**
	ATM mediated response to DNA double-strand break					**1**
	Gap-filling DNA repair synthesis and ligation in GG-NER					**1**
	Gap-filling DNA repair synthesis and ligation in TC-NER					**1**
	Global genomic ner (gg-ner)					**1**
	Inhibition of replication initiation of damaged DNA by Rb/E2F1					**1**
	Lagging strand synthesis					**1**
	Leading strand synthesis					**1**
	Nucleotide excision repair					**1**
	Processive synthesis on the lagging strand					**1**
	Removal of the Flap Intermediate from the C-strand					**1**
	Repair synthesis for gap-filling by DNA polymerase in TC-NER					**1**
	Transcription-coupled NER (TC-NER)					**1**
Lipid metabolism	Type I hemidesmosome assembly	**1**				
	Fatty acid activation			**1**		
	Fatty Acyl-coa Biosynthesis	**1**				
	Triglyceride biosynthesis	**1**				
Immune response	Formyl peptide receptors bind formyl peptides and many other ligands			**1**		
	Leukotriene receptors				**1**	
	Platelet aggregation (plug formation)			**1**		
NA+ influx	Unblocking of NMDA receptor, glutamate binding, and activation			**1**		
Signaling pathways	Negative regulators of RIG-I/MDA5 signaling					**1**
	Mevalonate pathway I	**1**				
	Signaling events mediated by HDAC Class III	**1**				
	Attachment of GPI anchor to upar	**1**				
RNA ploymerase I activity	Rna polymerase i chain elongation	**1**				
	Rna polymerase i promoter clearance	**1**				
	Rna polymerase i promoter opening	**1**				
	Rna polymerase i transcription	**1**				
Telomere maintenance pathways	Extension of Telomeres					**1**
	Polymerase switching on the C-strand of the telomere					**1**
	Processive synthesis on the C-strand of the telomere					**1**
	Telomere C-strand (Lagging Strand) Synthesis					**1**
	Telomere C-strand synthesis initiation					**1**
	Packaging of telomere ends	**1**				
Thiosulfate disproportionation III	Thiosulfate disproportionation III (rhodanese)					**1**

Telomere shortening has been reported in sulfur mustard-exposed Iranian veterans, and this phenomenon is correlated with oxidative stress biomarkers.^22^, ^23^ Additionally, changes in genes associated with telomere maintenance have been associated with various hazardous materials and toxic agents, including Benzene.^24^, ^25^ Furthermore, altered expression of genes related to telomere length regulation has been observed in certain smokers.^26^

In line with these findings, our results demonstrate the involvement of genes associated with “Packaging of Telomere Ends” and “Telomere Maintenance” pathways in smokers as well. However, in the case of acute mustard exposure, we observed different pathways related to telomer maintenance, namely “Extension of Telomeres”, “Polymerase switching on the C-strand of the telomere”, “Processive synthesis on the C-strand of the telomere”, and “Telomere C-strand (Lagging Strand) Synthesis”. Similarly, we did not observe any telomere-related signaling terms in the context of mechanical injury, COPD, and Mustard Lung. It is important to note that an imbalance of DNA injury repair processes can contribute to lung carcinogenesis in both acute and chronic states of sulfur mustard exposures^27^, ^28^, ^29^, ^30^ as well as in smokers.^31^

These findings highlight the critical role of telomere biology and maintenance in both sulfur mustard exposure and smoking-related conditions. The differences observed in specific gene expression patterns related to telomere dynamics between acute mustard exposure and smoking indicate distinct cellular responses to these different insults. Understanding the mechanisms underlying telomere dynamics in the context of toxic exposures can provide valuable insights into the development of lung-related diseases and potentially guide the development of targeted therapeutic interventions. Furthermore, these findings emphasize the importance of maintaining proper DNA repair mechanisms to prevent the onset of lung cancer. The absence of telomere-related signaling terms in mechanical injury, COPD, and Mustard Lung suggests the involvement of alternative mechanisms in the pathogenesis of these conditions. Further research is required to unravel these mechanisms and identify potential therapeutic targets for lung-related diseases associated with DNA damage and repair imbalances.

We have observed variations in physical cell-cell interactions among the different states we studied. Specifically, the pathways “c-src mediated regulation of Cx43 function and closure of gap junctions”, “p130Cas linkage to MAPK signaling for integrins”, and “Regulation of gap junction activity” were only identified in the mechanical injury of epithelial cells. Additionally, certain pathway terms were specific to the smoke and COPD states. Interestingly, we did not identify any unique cell-cell physical interaction term in the Mustard groups.

The regulation of physical cell-cell interaction is critical in the resolution of injuries during processes such as MET and EMT. We observed the involvement of MET and EMT signaling in all five conditions studied, albeit with different expression patterns. However, these pathways exhibited activity in the mustard groups (Supplementary Files 4 and 5). Numerous reports have highlighted the involvement of EMT in smokers, COPD patients, smokers, and lung cancer.^32^, ^33^, ^34^, ^35^ In COPD and smokers, we also observed the presence of MET related genes. The distinct expression patterns in MET and EMT in different studied groups may contribute to tissue remodeling in small airways. The balance between EMT and MET transitions is crucial for tissue repair and regeneration. This imbalance may help explain the persistent and inefficient tissue repair in Mustard Lung and COPD patients. Furthermore, the imbalance between MET and EMT has been associated with various lung cancers.^36^, ^37^ Additionally, we report distinct involvement of Notch signaling in tissue repair distinct the examined states. Notch signaling is associated with EMT activation and is also implicated in lung cancer.^38^

The findings from our analysis reveal interesting patterns of gene expression in different stages and etiologies of epithelial injury. In the context of EMT, several genes show distinct upregulation or downregulation patterns in smoke exposure and COPD compared to the other groups. FN1 and SERPINE1 are upregulated in smoke exposure and COPD, suggesting their potential involvement in the development and progression of these conditions. VCAN, on the other hand, is downregulated in smoke exposure and COPD, indicating its potential role in maintaining epithelial integrity. The upregulation of CCL2 specifically in COPD highlights its unique association with this condition.

When analyzing mechanical injury, specific genes such as GJA1, SPARC, COL6A1, and FBLN1 show upregulation in the later stages of the healing process. This suggests their potential involvement in tissue remodeling and repair following mechanical epithelial injury. The upregulation of CD163 in both COPD and mechanical injury indicates its role in the inflammatory response common to these conditions. SNAI2, upregulated exclusively in mechanical injury, may play a role in the wound healing process. FBN1 and CAV1 show differential upregulation patterns across different stages of mechanical injury, suggesting their involvement in specific phases of tissue repair.

The comparison of acute and chronic mustard exposure reveals distinct gene expression patterns. WWTR1 is upregulated in both stages, indicating its potential role in the transition from acute to chronic mustard exposure. The upregulation of FBN1, CAV1, SACS, PLN1, and SOBP specifically in the chronic mustard group suggests their involvement in the progression and chronicity of mustard-induced epithelial injury. Conversely, ITM2A is upregulated only in the acute mustard exposure group, indicating its specific role in the acute response to mustard.

In the context of MET, several genes exhibit differential expression patterns between acute and chronic stages of mustard exposure. AGR2, CBLC, and CEACAM5 show decreased expression specifically in the acute state, suggesting their potential roles in the acute response to mustard. FXYD3, CLDN7, and IRF6, on the other hand, are upregulated only in the acute state, indicating their involvement in the early stages of epithelial repair.

Comparison of smoke exposure and COPD groups reveals upregulation of several genes after smoke exposure. ERBB2, CLDN7, ERBB3, CBLC, EPCAM, IRF6, PLLP, and C1orf116 are all upregulated, suggesting their potential involvement in the pathogenesis of smoke-induced epithelial injury. The upregulation of CEACAM5 in both smoke exposure and COPD indicates its potential role as a common marker or mediator in these conditions.

Furthermore, the specific downregulation of AKR1B10 and TFF1 in the chronic mustard group, along with the upregulation of SHANK2, provides insights into the potential roles of non-hub genes in the chronic state of mustard exposure and associated epithelial injury.

Research has identified various biological pathways associated with lung diseases, but these studies often focus on individual conditions without comprehensive comparisons across different types of exposures. This analysis uniquely examines both acute and chronic states of COPD, Mustard Lung, and exposures to sulfur mustard and cigarette smoke, revealing distinct and shared biological pathways. Notably, the identification of unique DNA repair pathways activated by sulfur mustard exposure, which are absent in Mustard Lung or mechanical injury, highlights a significant finding that adds depth to our understanding of the molecular responses to different lung insults. The analysis also uncovers specific telomere maintenance pathways in smokers, contrasting with the pathways observed in acute mustard exposure, indicating distinct cellular responses to these environmental stressors.

Finally, we acknowledge that mechanical wound healing differs from chemical injury repair in several important respects. Chemical injuries, such as those induced by sulfur mustard or cigarette smoke, involve additional pathogenic mechanisms including DNA alkylation, oxidative stress, and prolonged inflammatory responses that are not present following mechanical injury alone. Therefore, the mechanical injury dataset used in this study should be interpreted as a model of baseline or ‘unperturbed’ epithelial repair, rather than as a direct surrogate for chemical injury. This approach follows established precedent in the literature, where mechanical injury has been used to characterize core repair transcriptomes and to isolate the effects of chemical exposures on repair processes. Future studies directly comparing mechanical and chemical injury models will be valuable to further delineate injury-specific repair mechanisms.

## 5 Conclusion

In conclusion, this study provides valuable insights into the critical role of lung epithelial cells in the development of acute and chronic lung injuries resulting from exposure to mustard gas and cigarette smoke. The findings highlight the remarkable ability of these cells to respond to damage, as evidenced by the activation of key pathways involved in DNA repair, cellular transformation, and tissue remodeling. Understanding the intricate mechanisms governing the interaction between epithelial cells and environmental factors holds immense potential for the development of targeted therapeutic strategies aimed at mitigating the devastating consequences of lung injuries. These findings lay the groundwork for future research endeavours, paving the way for improved interventions and better outcomes for individuals affected by these complex conditions. Therapeutic strategies aimed at modifying the devastating consequences of lung injuries. The identification of specific hub genes with high centrality in the EMT and MET networks (e.g., FN1, SERPINE1) highlights novel potential therapeutic targets and the translational relevance of these findings in managing acute and chronic lung diseases. While our multi-cohort transcriptomic meta-analysis and network-based approach provide robust in silico predictions of candidate hub genes, the findings remain computational in nature. Therefore, further experimental validation—such as qRT-PCR, Western blotting, or immunohistochemistry in appropriate in vitro or in vivo models—is necessary to confirm the functional relevance and expression patterns of the prioritized genes prior to any clinical translation.

## 6 Limitations

Several limitations of this study should be acknowledged. **First**, the analysis is entirely computational (in silico) and relies exclusively on publicly available transcriptomic datasets. Consequently, the identified genes, pathways, and network hubs represent correlative associations rather than causal relationships. No functional validation experiments—such as CRISPR-Cas9 gene editing, siRNA knockdown, airway organoid models, or in vivo studies—were performed. Therefore, the proposed roles of specific genes in airway epithelial repair, EMT/MET balance, and tissue remodeling remain hypothetical and require experimental validation.

**Second**, the analyzed datasets originate from different microarray platforms, cell types, and experimental designs, including primary bronchial epithelial cells, small airway epithelial cells, endobronchial biopsies, and the A549 cell line. These datasets represent complementary biological models rather than directly comparable sample types. To minimize technical bias, each dataset was analyzed independently and cross-study integration was performed only at the level of overlapping genes and pathway enrichment results rather than by merging expression matrices. Nevertheless, this biological and technical heterogeneity may influence the comparability of transcriptional responses across studies.

**Third**, because several datasets contained relatively small sample sizes, applying stringent multiple-testing corrections substantially reduced statistical power and resulted in very few detectable genes. Therefore, raw *p*-values were used at the initial DEG screening stage together with a biological effect-size threshold (|fold change| ≥ 2). Although additional filtering and cross-dataset comparisons were applied to prioritize robust signals, this strategy may increase the risk of type I errors.

**Fourth**, because stringent multiple-testing corrections substantially reduced statistical power in small datasets, raw p-values were used at the initial DEG screening stage together with a biological effect-size threshold (|fold change| ≥ 2). Although additional filtering and cross-dataset comparisons were applied to prioritize robust signals, this strategy may increase the risk of type I errors.

**Fifth**, the analyzed datasets are derived from bulk epithelial samples, which may mask cell-type–specific transcriptional responses. Single-cell RNA sequencing approaches would be required to resolve heterogeneity among airway epithelial subpopulations during injury and repair.

**Finally**, the present analysis focuses on transcriptional regulation and does not capture post-transcriptional mechanisms, post-translational modifications, protein stability, regulatory non-coding RNA networks, or phenotypic transitions that may influence epithelial repair processes. Therefore, the findings should be interpreted as exploratory and hypothesis-generating, providing candidate genes and pathways for future mechanistic and experimental studies of airway injury and repair.

## CRediT authorship contribution statement

**Masoud Arabfard:** Writing – original draft, Visualization, Supervision, Methodology, Formal analysis, Data curation. **Zahra Salehi:** Writing – original draft, Validation. **Mostafa Ghanei:** Writing – review & editing, Project administration, Investigation. **Sadegh Azimzadeh Jamalkandi:** Writing – review & editing, Visualization, Supervision, Project administration, Methodology, Investigation, Conceptualization.

## Declaration of competing interest

The authors declare that they have no known competing financial interests or personal relationships that could have appeared to influence the work reported in this paper.

## Appendix A Supplementary data

Supplementary material 1: Including analysis parameters including for DEGs for all conditions.

Supplementary material 2: Pairwise comparison of DEGs between conditions using venn diagrams.

Supplementary material 3: Comprehensive lists of significantly enriched pathways for each dataset, with associated statistics.

Supplementary material 4: Lists of genes linked to Epithelial-Mesenchymal Transition (EMT) pathways for each disease state.

Supplementary material 5: Lists of genes linked to Mesenchymal-Epithelial Transition (MET) pathways for each disease state.

Supplementary material 6: Identifies gene clusters and key hub genes within EMT pathways for network and heatmap construction.

Supplementary material 7: Identifies gene clusters and key hub genes within MET pathways for network and heatmap construction.

Supplementary material 8: Specifies thresholds and parameters used for MET and EMT network construction and analysis.

Supplementary material 9: Data table detailing relationships between genes, pathways, and conditions which is used for network reconstruction.

Supplementary material 10: Visual representation of interconnected genes, pathways, and conditions.
