# Supplementary figures and images for: Transcriptomic landscape of airway epithelial repair: Contrasting acute and chronic injury in mustard lung and COPD
Source: J Genet Eng Biotechnol. 2026 Jun 24;24(3):100756. doi: 10.1016/j.jgeb.2026.100756 (PMC13320475; doi:10.1016/j.jgeb.2026.100756)

### a) Downregulated genes

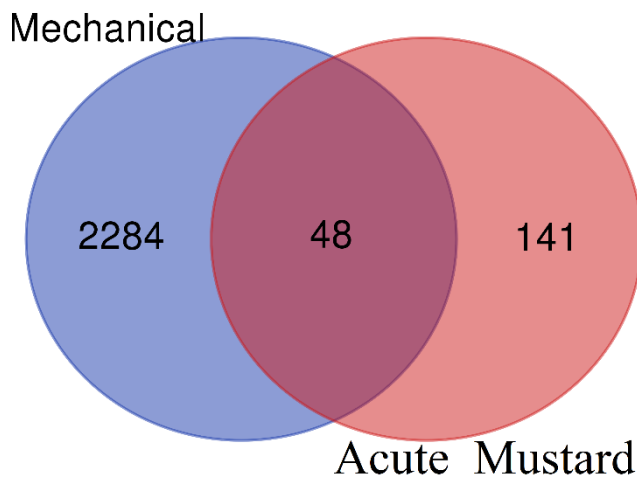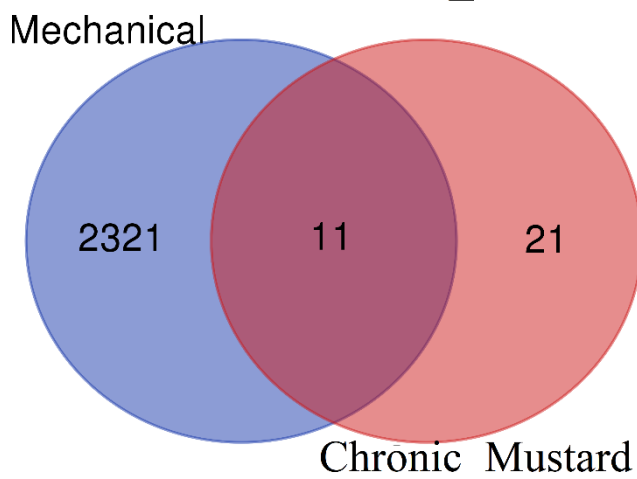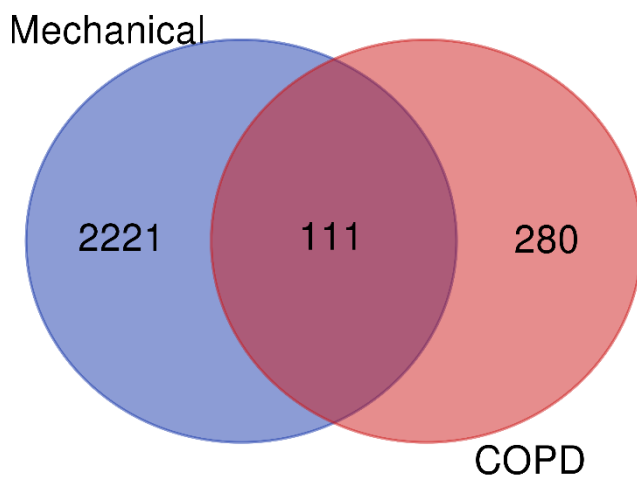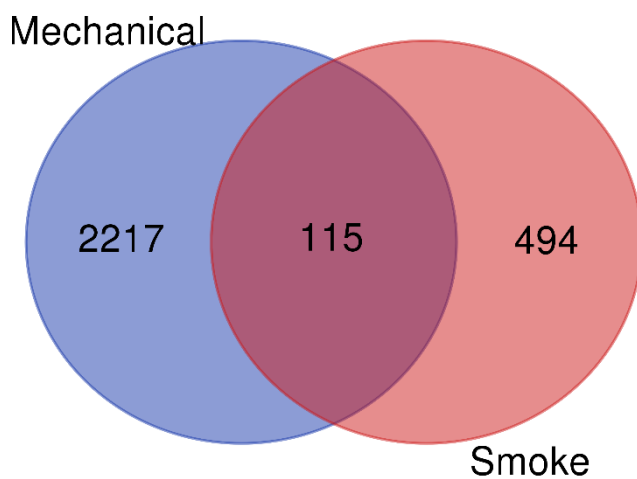

### b) Upregulated genes

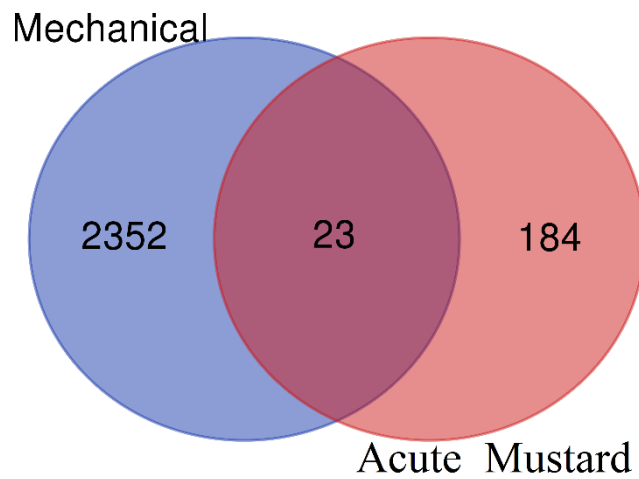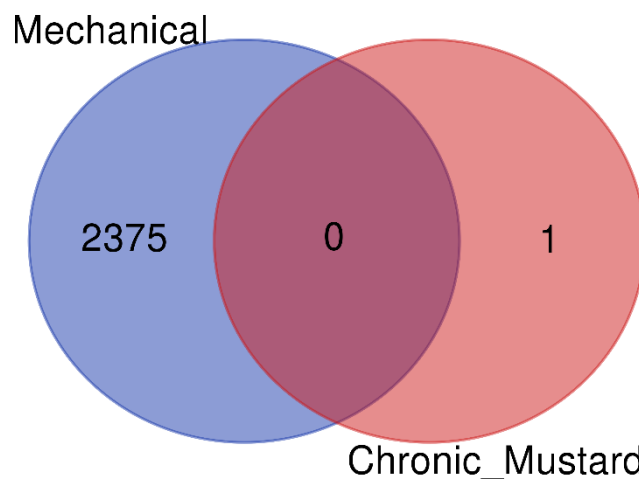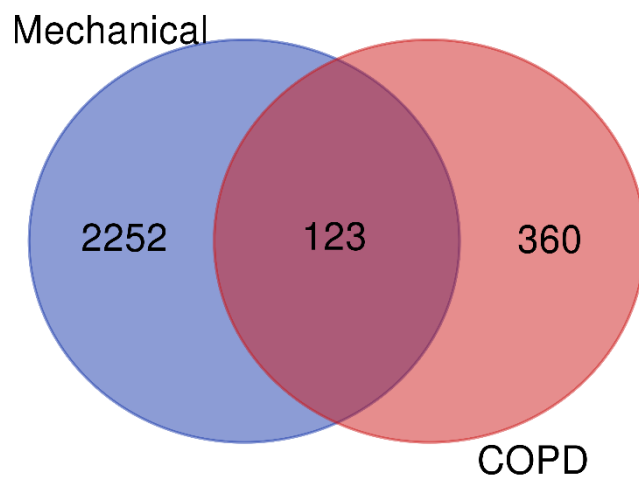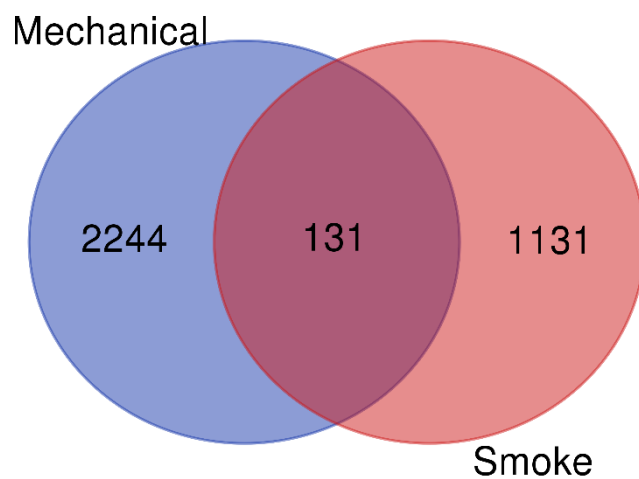

Supplement: Supplementary file 2 — Supplementary material 2: Pairwise comparison of DEGs between conditions using venn diagrams. [file mmc2.pdf]

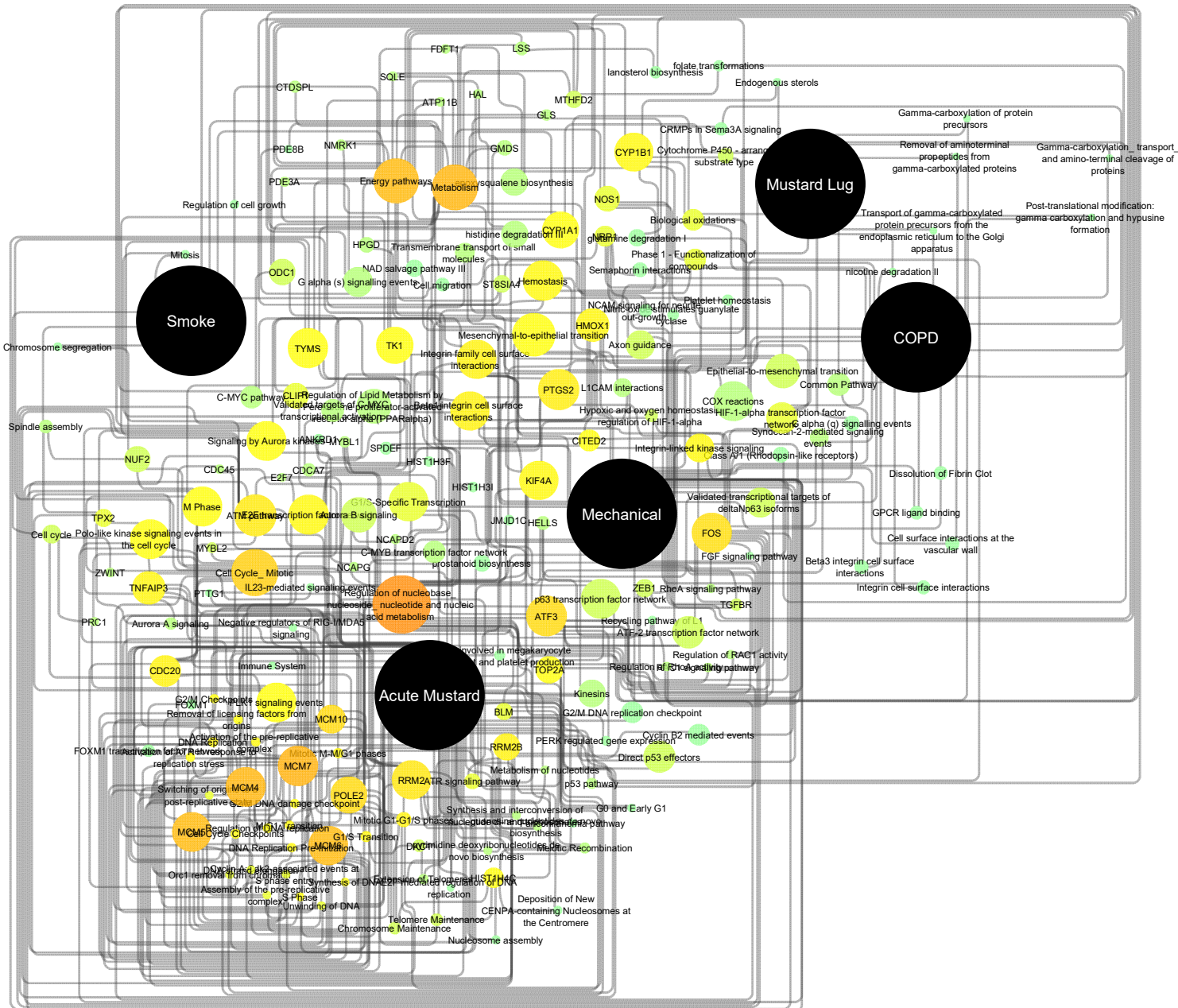

Supplement: Supplementary file 10 — Supplementary material 10: Visual representation of interconnected genes, pathways, and conditions. [file mmc10.pdf]
