# Supplementary material for: Transcriptomic landscape of airway epithelial repair: Contrasting acute and chronic injury in mustard lung and COPD
Source: J Genet Eng Biotechnol. 2026 Jun 24;24(3):100756. doi: 10.1016/j.jgeb.2026.100756 (PMC13320475; doi:10.1016/j.jgeb.2026.100756)
Supplement: Supplementary file 4 — Supplementary material 4: Lists of genes linked to Epithelial-Mesenchymal Transition (EMT) pathways for each disease state. [file mmc4.docx]

**EMT involved genes**

| Mustard Lung_EMT | Acute mustard_EMT | Mechanical injury_EMT | COPD_EMT | Smoke_EMT |
| --- | --- | --- | --- | --- |
| CDH2 | RGS2 | CCL2 | CCL2 | CCL2 |
| CYP1B1 | GLIPR1 | FN1 | FN1 | FN1 |
|  | LY96 | SERPINE1 | SERPINE1 | SERPINE1 |
|  | CRYAB | AP1S2 | CDH2 | AP1S2 |
|  |  | AKT3 | COL6A1 | AKT3 |
|  |  | BICC1 | DPYSL3 | BICC1 |
|  |  | CAV1 | FBN1 | CAV1 |
|  |  | LOXL2 | ITM2A | LOXL2 |
|  |  | MPDZ | KIAA1462 | MPDZ |
|  |  | TRPC1 | MRC1 | TRPC1 |
|  |  | ZEB1 | MYLK | ZEB1 |
|  |  | DSE | PLN | DSE |
|  |  | VCAN | PMP22 | VCAN |
|  |  | CDH2 | SOBP | CYP1B1 |
|  |  | COL6A1 | CYP1B1 | AKAP12 |
|  |  | DPYSL3 | AKAP12 | MAP1B |
|  |  | FBN1 | MAP1B | SLC2A3 |
|  |  | ITM2A | SLC2A3 | TPM2 |
|  |  | KIAA1462 | TPM2 | CRYAB |
|  |  | MRC1 | NAP1L3 | CEP170 |
|  |  | MYLK | PDGFC | CLIC4 |
|  |  | PLN | SLIT2 | EMP3 |
|  |  | PMP22 | UCHL1 | GEM |
|  |  | SOBP | ZFPM2 | GFPT2 |
|  |  | RGS2 |  | SACS |
|  |  | GLIPR1 |  | TUBA1A |
|  |  | LY96 |  | CTSK |
|  |  | BAG2 |  | FSTL1 |
|  |  | CALD1 |  | C1S |
|  |  | CD163 |  | NR3C1 |
|  |  | CDH11 |  | GREM1 |
|  |  | CLEC2B |  | MOXD1 |
|  |  | COL14A1 |  | WWTR1 |
|  |  | COL15A1 |  |  |
|  |  | COL6A2 |  |  |
|  |  | COLEC12 |  |  |
|  |  | CRISPLD2 |  |  |
|  |  | CSF2RB |  |  |
|  |  | CSRP2 |  |  |
|  |  | CXCL13 |  |  |
|  |  | CXCR4 |  |  |
|  |  | F13A1 |  |  |
|  |  | FAP |  |  |
|  |  | FBLN1 |  |  |
|  |  | FHL1 |  |  |
|  |  | GJA1 |  |  |
|  |  | GLYR1 |  |  |
|  |  | GUCY1B3 |  |  |
|  |  | IGFBP5 |  |  |
|  |  | ISLR |  |  |
|  |  | LGALS1 |  |  |
|  |  | MAF |  |  |
|  |  | MAFB |  |  |
|  |  | MS4A4A |  |  |
|  |  | MS4A6A |  |  |
|  |  | MYL9 |  |  |
|  |  | PTGIS |  |  |
|  |  | QKI |  |  |
|  |  | RECK |  |  |
|  |  | SFRP1 |  |  |
|  |  | SNAI2 |  |  |
|  |  | SPARC |  |  |
|  |  | SPARCL1 |  |  |
|  |  | SRGN |  |  |
|  |  | STON1 |  |  |
|  |  | SYNE1 |  |  |
|  |  | TNS1 |  |  |
|  |  | TUBB6 |  |  |
|  |  | VSIG4 |  |  |
|  |  | ZEB2 |  |  |
|  |  | ANK2 |  |  |
|  |  | ASPN |  |  |
|  |  | IFFO1 |  |  |
|  |  | TAGLN |  |  |
|  |  | TGFB1I1 |  |  |
