# Supplementary material for: Transcriptomic landscape of airway epithelial repair: Contrasting acute and chronic injury in mustard lung and COPD
Source: J Genet Eng Biotechnol. 2026 Jun 24;24(3):100756. doi: 10.1016/j.jgeb.2026.100756 (PMC13320475; doi:10.1016/j.jgeb.2026.100756)
Supplement: Supplementary file 5 — Supplementary material 5: Lists of genes linked to Mesenchymal-Epithelial Transition (MET) pathways for each disease state. [file mmc5.docx]

**MET involved genes**

| Mustard Lung_MET | Acute mustard_MET | Mechanical injury_MET | COPD_MET | Smoke_MET |
| --- | --- | --- | --- | --- |
| HPGD | IL1RN | IL1RN | IL1RN | S100P |
| TFF3 | KRT15 | KRT15 | S100P | GMDS |
| PSCA | CEACAM1 | CEACAM1 | GMDS | GDF15 |
|  | S100P | HPGD | AKR1B10 | GPRC5A |
|  | GMDS | SCEL | GPX2 | HPGD |
|  | AKR1B10 | SPDEF | TFF3 | SCEL |
|  | GPX2 | EPB41L4B | SCEL | SPDEF |
|  | GDF15 | HNMT | SPDEF | CEACAM5 |
|  | GPRC5A | SFN | EPB41L4B | ALOX5 |
|  | IRF6 | UGT1A1 | HNMT | AREG |
|  | LCN2 | CEACAM5 | SFN | TFF1 |
|  | PRSS8 | ALOX5 | UGT1A1 | AQP3 |
|  | RAB11FIP1 | AREG | TFF1 | CD2AP |
|  | CLDN7 | BSPRY | ABCC3 | CEACAM7 |
|  |  | CBLC | AGR2 | CKMT1A |
|  |  | COMT | AZGP1 | ERBB2 |
|  |  | DSC2 | CEACAM6 | EXPH5 |
|  |  | HES1 | CYP4F3 | MYO1D |
|  |  | PHLDA2 | FUT3 | OAS1 |
|  |  | SHANK2 | NQO1 | PLLP |
|  |  | TTC39A | VGLL1 | PRR15L |
|  |  | EZR |  | S100A14 |
|  |  | GALNT3 |  | SLC22A18 |
|  |  | KCNK1 |  | TOX3 |
|  |  | KRT7 |  | TSPAN1 |
|  |  | RAB20 |  | TSPAN13 |
|  |  | RBM47 |  | TSPAN8 |
|  |  | TMC6 |  | TUFT1 |
|  |  |  |  | ITGB6 |
|  |  |  |  | TSPAN15 |
|  |  |  |  | ANXA4 |
|  |  |  |  | TGFA |
|  |  |  |  | POF1B |
|  |  |  |  | MYO6 |
|  |  |  |  | PLS1 |
|  |  |  |  | FA2H |
|  |  |  |  | GALE |
|  |  |  |  | B3GNT3 |
|  |  |  |  | BCAS1 |
|  |  |  |  | C1orf116 |
|  |  |  |  | EPCAM |
|  |  |  |  | ERBB3 |
|  |  |  |  | ESRP1 |
|  |  |  |  | FXYD3 |
|  |  |  |  | TRIM31 |
